# National quality and performance system for Divisions of General Practice: early reflections on a system under development

**DOI:** 10.1186/1743-8462-5-8

**Published:** 2008-05-30

**Authors:** Karen L Gardner, Beverly Sibthorpe, Duncan Longstaff

**Affiliations:** 1 Australian Primary Health Care Research Institute, Australian National University, Canberra, ACT, Australia; 2 Menzies School of Health Research, Darwin, NT, Australia

## Abstract

**Background:**

Governments are increasingly introducing performance management systems to improve the quality and outcomes of health care. Two types of approaches have been described: assurance systems that use summative information for external accountability and internally driven systems that use formative information for continuous quality improvement. Australia recently introduced a National Quality and Performance System (NQPS) for Divisions of General Practice that has the dual purposes of increasing accountability and improving performance. In this article, we ask whether the framework can deliver on its objectives for achieving accountability and fostering performance improvement. We examine the system in terms of four factors identified in a recent systematic review of indicator systems known to improve their use. These are: involving stakeholders in development; having clear objectives; approach to data collection and analysis including using 'soft data' to aid interpretation; and feeding back information.

**Results:**

We found that early consultative processes influenced system development. The system promotes the collection of performance information against defined program objectives. Data includes a mix of qualitative and quantitative indicators that are fitted to a conceptual framework that facilitates an approach to performance assessment that could underpin continuous quality improvement at the Division level. Feedback of information to support the development of quality improvement activities has not been fully developed.

**Conclusion:**

The system currently has elements that, with further development, could support a more continuous quality improvement or assurance based approach. Careful consideration needs to be given to the development of methods for analysis and review of performance indicators, performance assessment and engagement with consumers. The partnership arrangement that supported early development could be expected to serve as an important vehicle for further development.

## Background

Performance management systems are increasingly being introduced to improve the quality and performance of healthcare. They are the latest innovation in a raft of technologies that seek to improve the accountability of healthcare providers in ways that stimulate improvements in the delivery of care [1]. In the primary health care sector, such systems involve the use of strategies aimed at improving the performance of Primary Care Organisations as well as the quality of care provided in general practice. They typically comprise: a set of performance indicators designed to highlight variations in care; a report card system for publishing performance data that is aimed at providing consumers and purchasers with information they need to make more informed purchasing decisions; and a set of mechanisms aimed at motivating behaviour change amongst professionals with capacity to drive improvements [1].

Introduction of these systems into the public sector marks a policy shift toward greater management of primary care and a move away from more traditional command and control approaches that monitor service cost, activity and output data to more indirect and hands off regulatory approaches that measure performance against specified targets [2]. In this way, performance management systems provide a link between central control and local responsibility [3,4], fulfilling functions related to promoting both accountability to government and continuous quality improvement (CQI) at the organisational level.

Two types of performance systems have been described: those that use summative mechanisms for the purpose of achieving external accountability such as to funding bodies and more internally driven systems that use formative mechanisms for the purpose of achieving continuous quality improvement [2]. Both systems use measurement and benchmarking techniques to identify variation in performance but they have different philosophical bases and emphases and use data in different ways to promote behaviour change. Assurance systems tend to rely on ranking processes and the development of league tables to establish levels of performance, linking these to rewards and sanctions whereas CQI systems tend to use statistics to make comparisons descriptively, utilising more informal benchmarking processes as a starting point for identifying important issues and engaging the stakeholders in dialogue to generate insights into practice [2]. These different emphases have implications for the type of data required, the choice of methods used for analysis and the types of mechanisms used to promote behaviour change.

### Effectiveness

Considerable debate exists about the value of performance systems and evidence of their effectiveness in improving the quality and outcomes of care is scant, particularly in primary care settings [5]. That which does exist comes mostly from studies of assurance systems in hospitals, in the context of debate about the relative merits of publishing performance data. Such studies have examined attitudes to the public release of comparative data on performance [6] and its impact on the behaviour of health care purchasers, consumers and providers [7-10]. There is some evidence that provider organizations are more likely than consumers, physicians or purchasers to respond to report cards [11] and hospitals have been shown to be willing to utilise performance data for internal quality improvement activities [5]. Such actions have also been associated with improvements in the processes and outcomes of care [12]. At the same time, however, the publication of performance data has been associated with a host of unintended consequences, perverse incentives and technical problems [13] that suggest significant ambiguity about the potential for its use in promoting improvement and change [14]. There are also suggestions that impacts, even on providers, tend to wane, as the mechanisms employed for altering consumer and purchasing behaviour may produce little impact over time [15]. Given this uncertainty, three authors have argued in favour of a focus at the "soft end" of performance management, namely on the development of Continuous Quality Improvement (CQI) approaches that encourage performance improvement through reflection, feedback and learning [2,5,7].

### National Quality and Performance System for Divisions of General Practice

The Australian Government introduced a National Quality and Performance System for Divisions of General Practice in March 2005. It is an ambitious performance management framework that has the dual purposes of increasing accountability and improving performance, the latter explicitly based on the ideal of CQI. NQPS stated aims are to establish "a process to reward high performance, promote best practice, support under performance and sharpen the focus of the network in order to ensure all communities can have similarly high expectations of Divisions network members" (pp2.9) [16]. The system is still under development and important decisions are yet to be made in relation to operationalising the key elements. How this occurs is likely to impact on the extent to which the framework is weighted in favour of CQI or accountability. In this article, we ask whether the framework can deliver on its initial aims of achieving accountability and fostering performance improvement. To do this, we examine the system in terms of four factors identified in a recent systematic review of indicator systems [2] as being associated with their improved use. These are: involving stakeholders in development; having clear objectives; approach to data collection and analysis including using 'soft data' to aid interpretation; and feeding back information.

## Methods

The study has two components – a review of published theoretical and empirical studies of the use of performance management systems that examined the evidence relating to the role, function and effectiveness of primary care performance management systems; and analysis of NQPS development and implementation processes. The latter was facilitated by our involvement in key aspects of development of the system, including development of the underpinning conceptual framework [17] and leadership of the process used to develop the indicators through a contract between the Australian Government Department of Health and Ageing and the Australian Primary Health Care Research Institute. We have used the four factors identified in the systematic review described above as our framework for examining the extent to which this system can deliver on its initial aims and to consider how key system components might be further developed to best achieve its objectives. For each of the four factors we have woven together the evidence from the literature and the results of our analysis of the development and implementation of the NQPS which draws on some previously published work [18].

## Discussion

### Setting

Divisions of General Practice are local networks of general practitioners operating in defined geographical areas to improve health. They were established by the Australian Government in 1992 to encourage GPs to work together and form links with other health professionals to upgrade the quality of health service delivery at the local level. There are now 119 Divisions across Australia, their state based organisations (SBOs) and a national peak body, the Australian General Practice Network (AGPN) which together comprise the Divisions network. Divisions receive core funding from the Australian Government Department of Health and Ageing (DoHA) and are governed by elected boards whose members are predominantly GPs. They are a diverse group, both in terms of their numbers of constituent GPs which range from eight to over 300, and in terms of the characteristics and size of the communities and populations they serve which range from under 17,000 people in some rural and remote regions to almost 600, 000 in metropolitan areas. Ninety-five percent of Australian GPs are now members of Divisions [19].

The NQPS is a key component of the Australian Government's response to a 2003 Review of the Role of Divisions of General Practice. It found a lack of clarity regarding government expectations of the network's performance, variability in performance and little capacity in the program to demonstrate achievements and value for money. The Review recommended that Divisions should have clearer goals, clarity of roles, increased accountability for outcomes and taxpayers' funding, improved consistency of performance and governance across the network, greater alignment with territory and state boundaries and an increased focus on the delivery of services [20].

### National Quality and Performance System – description of key components

The NQPS comprises three key mechanisms for achieving accountability and quality improvement. These are a set of national performance indicators, a requirement for accreditation and a process of performance assessment that is linked to a system of rewards and sanctions. Table 1 below summarises the key components.

**Table 1:** NQPS key components

	Components and processes
National Performance Indicators	52 National indicators spanning government priorities
	• Governance
	• Prevention and early intervention,
	• Access
	• Integration
	• Chronic disease management
	Compulsory reporting against a subset of national indicators and choice in reporting against local programs
	PIs underpinned by a conceptual framework and technical details 4 levels of indicators from processes through to client outcomes
Accreditation	Requirement for all Divisions to become accredited by June 2008
	Once accredited, Divisions no longer required to report on governance indicators
Performance Assessment	Performance against indicators is to feed into individual performance appraisal
	At the national level, analysis and benchmarking support comparison and provide an aggregated picture of performance
	Links to rewards (still to be determined) to reward high performance and support improvement

As shown, fifty-two national performance indicators (NPIs) capture government expectations for performance in relation to governance, prevention and early intervention, access, integration and chronic disease management. Divisions are required to report against a compulsory subset of these indicators. There was choice in the number and level of indicators to be reported in the first year, as well as scope for reporting achievements against locally conducted programs although this was not compulsory. Once Divisions are accredited by an approved provider, they will no longer be required to report against the majority of the governance indicators. The first full year of reporting was completed in September 2006.

The NPIs are underpinned by a conceptual framework [17] that is based on Donabedian's now classic structure, process, outcome model for assessment of quality of care [21] and supported by a set of technical details. The technical details make provision for the collection of explanatory text that is designed to provide important contextual information that may influence assessment of performance.

Assessment of performance will be conducted at two levels: at the network level, where benchmarking can facilitate comparison between Divisions and at the Division level, where individual achievement against NPIs will attract a score that feeds into a process of overall performance appraisal. This latter process includes consideration of achievement relating to contractual obligations, local programs, links within the network and organisational capacity. Assessment against NPIs may be augmented in future with the determination of targets.

Performance was initially intended to link to a set of rewards and sanctions [22]. Those mooted included 'earned autonomy', which included reduced reporting requirements for high performers, development support for those not performing to expectation, as well as competitive access to additional funds for Divisions shown to have particular strengths in areas of work that could support improvement and build capacity in the network, through the establishment of a Performance and Development Funding Pool. This pool was implemented in the first year but not thereafter; earned autonomy has not been implemented and consideration is currently being given to alternative methods for recognising and supporting performance. Sanctions were to have included mediation for Divisions identified as 'under-performing' but it is not clear what form this would take.

Publication of performance data did not feature in early documentation about the system. Australia does not currently publish primary health care system performance information but the Australian Commission on Safety and Quality in Health Care (previously the Australian Council of Safety and Quality in Health Care) has signaled its intention to do so [23]. It is unclear whether this would extend to publication of Divisions' performance data at this stage.

### Involving stakeholders in development

Development and implementation of the NQPS was overseen by a Review Implementation Committee (RIC) established by the Australian Government Department of Health and Ageing. It comprised representatives from Government and the Divisions network who operated through working groups to oversee the development of the key system components as well as the administrative processes that were needed to support them. This included development of the NPIs, a strategy to support accreditation and development of the system for performance assessment. While much of this early work was conducted within extremely tight timeframes, it was also accompanied by processes of consultation which appeared to achieve the endorsement of key members of the Divisions network [18]. Despite this, various components of the NQPS framework have not subsequently been implemented and there has not yet been agreement on how the performance assessment system will be developed. Without this, the system cannot be fully operationalised.

At the outset, development of National Performance Indicators was contracted to two experts: a legal expert on governance who developed governance indicators in consultation with the network through a series of key meetings, and to a primary health care research organisation who engaged a group of national and international clinicians, consumers and academics to develop the clinical and other performance indicators which were then subject to processes of consultation with Divisions members and other stakeholders. A related working group was also established to drive the development of an automated reporting system for NPIs.

In a series of interviews we conducted in early 2006 with a representative sample of Division CEOs, cautious support for the concept of performance indicators was expressed but this was accompanied by widespread concern that clinical indicator data that would have to come from General Practice to report on some indicators would be difficult to obtain, both because many GPs would not support reporting of data to government and because of the capacity of existing data sources to provide the quality required. Some Division CEOs saw this as a major impediment that could not be addressed in the absence of formal agreements between Divisions, general practice and Government [18]. Other CEOs, whose Divisions already support general practices to collect such data and collate it on their behalf, have argued that the key to addressing these concerns lies in Divisions retaining ownership and management of the data at the local level with reporting to Government of agreed aggregate data. Freeman has argued that lack of clarity over the aims of an indicator system inevitably lead to problems over ownership of the data and disputes over their meaning and proper use [2]. While resistance is to be expected [24], these concerns were offset by the incremental approach to implementation that was being taken (which meant Divisions would have time to work toward reporting clinical indicators) and assurances that were also given that the system would be refined over time based on review and feedback, a matter that is considered important to ensure continued relevance [25]. This may be particularly important for Divisions in rural and remote areas whose priorities may not align as well with national programs as those of urban Divisions [18] and for the continued provision of local programs not currently captured under national performance indicators. Divisions subsequently established systems for the collection, reporting and analysis of performance indicator data [18] and by the end of 2006 a first year of performance data was submitted.

A consultant was also engaged to develop the strategic directions for the National Information Strategy which was to support the collection, analysis and feedback of data to Divisions [22]. A series of site visits, consultations and interviews with Divisions and other stakeholders was conducted and AGPN was subsequently contracted to provide the national coordination for a team of regional consultants who were employed to support Divisions in information management. The national information management strategy will be critical for addressing GP concerns about providing data to Divisions as well as providing assistance to support the development of IT systems and assurance processes that ensure quality, comparable data.

An accreditation working group was also established under the auspices of the RIC to assist Divisions to become accredited. Acting on the recommendations of the Working Group, the Department provided incentive payments to support Divisions to become accredited and all Divisions subsequently applied for and received the early adopter accreditation incentive payment [22]. By November 2006, 35 Divisions had achieved accreditation [22].

Division engagement in the development of the performance assessment system was through a Performance Working Group. While considerable work was undertaken to develop the specifications for assessing performance at the Division level and the detailed conceptual work relating to the reward system, which at that time was the Funding and Performance Pool and "earned autonomy", those concepts were later abandoned. More recently a decision has been taken to engage an AGPN/SBO Coalition to explore more "effective alternatives for encouraging high performance and supporting Divisions to raise their performance standard" [22] but concrete strategies have not yet been determined.

### Having clear objectives

From the outset, key government documents outlined clear objectives for the operation of the NQPS system. These were captured in the broad system aims referred to in early documents (see above) and the development of the key system components, which together reflected aspirations for improving accountability and fostering quality improvement. Indicators themselves have clear objectives but further articulation of the key objectives for analysing baseline data and identifying the processes for feeding back performance information to the sector are needed to operationalise the system as a whole and to achieve a shared understanding of the parameters and requirements for performance. Despite early involvement of the stakeholders in development, these further steps appear not to have occurred. Over time, this has amounted to a stagnation of the system and resulted in a drift away from the early emphasis on fostering quality improvement.

In relation to NPIs, clear objectives for performance are embedded in each of the domain areas for which indicator data is collected. Each major program area has a number of indicators that measure performance against defined program objectives. These were defined during the process of developing the indicators. In relation to diabetes for example, the objective is "Divisions will support general practices/GPs to provide optimal care and contribute to the achievement of the best possible health outcomes for patients with diabetes". Nine indicators across process and intermediate outcome domains measure performance based on best practice approaches to diabetes care [16]. This has several advantages, the first being that the approach is practical, explicit and grounded within funded program areas which makes sense to the various stakeholders [17]. It also facilitates alignment of the data requirements for external accountability with those of program activity within Divisions by providing information that is relevant in the organisational and clinical environments and which can underpin quality improvement processes. A number of CEOs reported having aligned their internal planning processes with reporting outcomes [18] such as in relation to reorganising roles and responsibilities in line with national directions for chronic disease management based on NPIs and in relation to the development of information systems that can support data capture. Realignment of duty statements and job descriptions, and the development of budget allocations to achieving outcomes were also mentioned.

At the network level, analysis of performance indicators is intended to provide a national picture of performance but the exact objectives and questions to be asked of baseline data have not been clearly articulated, at least in the public domain. This far, analysis of NPIs has been undertaken as part of the Government's processes of accountability to parliament for public money, as part of its program evaluation processes. A lack of clarity regarding the way in which data will be analysed and used to support a process of continuous quality improvement remains, despite early objectives for fostering continuous improvement.

### Approach to data collection and analysis, including use of 'soft data' to aid interpretation

While Governments use performance indicators to verify processes of quality improvement, providers need to be able to use the data to inform strategies for improving the quality of care. This requires achieving a balance between obtaining data that is of high enough quality and precision to ensure accuracy in measurement and capturing data that is relevant and of use in complex real world settings to underpin the development of strategies for improving care. Well derived performance indicators are not only markers of outcomes or processes that can be influenced by organisations but they should also act as a catalyst for change within organisations [26]. The NQPS has a number of important inbuilt features that potentially facilitate delivery of these important requirements.

NPIs include a mix of qualitative and quantitative indicators. These are fitted to a coherent conceptual framework [17] that explicitly identifies the processes of primary health care, reflecting attributes of the health system rather than attributes of the patient or other non-health care characteristics. Indicators are at four levels, from organisational processes through to intermediate outcomes and these are based on robust theory underpinning program evaluation more generally [17]. This allows Divisions at different stages of development to report performance and does not exclude those not yet able to report on intermediate outcomes. Over time, examination of the relationship between the four levels may provide an assessment of whether underpinning organisational structures and processes support client outcomes, thus potentially providing information that facilitates attribution of outcomes in ways that can inform the development of program activity. Considerable criticism of performance indicator systems revolves around their lack of capacity to examine attribution of outcomes and this is seen as a major impediment to efforts for quality improvement in the UK system for example [14].

Capture of contextual information to support interpretation of quantitative NPIs is a critical feature of the framework. This allows a Division to provide supporting information in relation to its performance in a particular area. This might include information relating to population characteristics or geographic location for example, where this may impact on the capacity of a Division to deliver programs or affect the likely uptake. This feature of the framework is designed to address difficulties associated with measurement where there is potential that confounding factors such as socio-economic variation, case mix, comorbidity and severity at the local level [27] may be responsible for observed variation. This is particularly pertinent for Australian Divisions because of the diversity of geographical and socio-demographic context between regions.

Availability and reliability of data are fundamental to any performance system, particularly when the key objective is to establish differences between groups [27]. The danger that between group differences are related to the quality of the data rather than to the quality of care is a real concern in systems like the NQPS where some indicators rely on routinely collected data from diverse general practices all over Australia. There was widespread concern amongst CEOs that the quality of chronic disease data in most general practices is not sufficiently robust to support performance assessment in the short term [18]. The national information management strategy is designed to play a key role in addressing this deficiency.

### Feed back of performance information

Systems that emphasise verification of performance tend to rely on ranking processes, using data in a summative fashion to establish certainty, linking results to rewards and sanctions. Systems emphasising quality improvement use data in a more formative way with a focus on providing information that can facilitate opportunities for reflection and learning. Early work on the development of the performance assessment system demonstrated a desire to capture the best of both systems – precision, rewards and sanctions and a capacity to use information to stimulate quality improvement. Further development of the system needs to build on this early work and clearly identify the purpose and processes of performance assessment.

Firstly, an early discussion in relation to performance assessment involved consideration of the appropriateness of ranking based on some summary score or rating. There were concerns that ranking would result in competitiveness within the network which would undermine the goal of strengthening the network as a whole, a stated priority in key Government documents. This is consistent with findings from studies of assurance systems which suggest that when providers are in competition to get good results, a range of perverse incentives and unintended consequences that undermine the conditions required to support quality improvement result [13]. In addition, the methods needed to fulfil requirements for certainty in ranking systems inevitably lead to technical problems and the use of complex statistical models that become so theoretical they tend to obscure transparency from the stakeholders who are the subject of such information and upon whom the system relies for improvements in care provision [5].

Other discussions around elements of the proposed performance assessment process signalled concern with the promotion of overall performance and development, rather than with simply verifying, rewarding and/or penalizing individual performance. Linking performance to rewards that seek to spread innovation across the sector, such as through a performance and development pool is one such example. Other discussions related to supporting individual improvement through setting targets for performance that would be negotiated by individual Divisions with their contract managers. At that time the Department intended to provide its project managers with training on continuous quality improvement approaches to ensure its performance assessment processes were underpinned by a continuous quality improvement philosophy [22].

When interviewed in 2006, CEOs expressed the view that performance assessment needed to be nationally consistent, a concern borne out of the fact that Divisions are managed by Commonwealth officers residing in different state offices and that consistency in management hitherto had not been evident. The introduction of a national system for measuring, comparing and monitoring performance alters the focus of attention to the national level and achieving consistency across the state offices assumes a greater level of importance. Other concerns related to the need for timely provision of performance feedback and an incremental approach to the development of targets for performance. Neither of these issues has since been resolved. In any case, they are elements only of a broader process that first needs to establish the key purpose for performance assessment. If a key purpose is to promote and foster quality improvement, then organisations need to be provided with the data so that it can inform the development of quality initiatives. To this end, there is evidence that multidisciplinary performance improvement teams are ideal for discussing and acting on identified problems [28].

## Conclusion

The NQPS is an ambitious framework that aims to promote both quality improvement and accountability. Government has inscribed its expectations for performance in the sector into its choice of performance indicators and these will provide the data against which a national picture of the network's achievements will be measured and individual performance assessed and compared. For the first time, a nationally consistent assessment of Divisions' capacity to improve health outcomes will be available. It's approach to linking the collection of Division level data to standardised program objectives, offers a capacity for supporting quality improvement that appears to be unique among comparator country frameworks.

The system currently contains something of a mix of summative and formative mechanisms that with further development could support a more CQI or assurance based approach. How the system refines and operationalises these components in relation to its priority questions and choice of methods for analyzing performance data, its approach to feeding back data to Divisions and putting into place a structure that supports the interpretation of data and identification of priorities for action (both at the individual Division and program levels) will determine its success as a performance framework. International evidence suggests that a focus on CQI is more likely to produce fewer technical indicator problems, greater levels of engagement with stakeholders and information that may provide a prompt and starting point for dialogue about improving quality.

The partnership arrangement that engaged the stakeholders and underpinned early development could be expected to serve as an important vehicle for further implementation, development and review. Three key areas need to be addressed. Firstly, the methods to be used for analysis, benchmarking, review and further development of the NPIs needs to be determined with a clear purpose in mind. This may best rest with a consortium of stakeholders who have the relevant sector, clinical and epidemiological knowledge to engage in a process of reflection that can identify national issues of concern and conduct review activities that will ensure that maximum use of performance information is made in relation to supporting quality improvement activities. Ongoing support for a national information strategy is critical to addressing current concerns in general practice and delivering data that can underpin this system.

Secondly, the partnership needs to work with the AGPN/SBO coalition to determine the purpose and parameters that will operate in the performance assessment process, the philosophy that will underpin it and the developmental supports that will be required to build capacity across the entire sector.

Thirdly, there is increasing recognition internationally of the need for consumers, as well as funders and providers, to have access to information about system performance and it seems timely for Australia to examine how this might best be done. Bringing consumers into the partnership arrangement at a high level may provide scope for further examination of how consumers might best be served by health performance management systems in the Australian context.

As it currently stands the NQPS has not realised its potential for achieving accountability and fostering quality improvement. Over time, its implementation has become more reminiscent of the traditional command and control approach to monitoring cost, activity and output data that has dominated contract management in recent years. As a regulatory framework that seeks to measure performance in terms of client outcomes and health improvement by linking accountability requirements with quality improvement processes, it still has some way to go.

## Competing interests

Two authors BS and KG developed the conceptual framework for performance assessment in primary health care that underpins the NQPS. BS was supported by DL in leading the team that was commissioned to develop the National Performance Indicators. KG worked in the Australian Government Department of Health and Ageing on aspects of development of the NQPS.

## Authors' contributions

KG developed the idea for the article and prepared early drafts, BS contributed to defining and shaping the arguments and added additional material for subsequent drafts, DL conducted literature searches associated with the article. All three authors read and approved the final draft.
